# Treatment of Hydrothermal-Liquefaction Wastewater with Crossflow UF for Oil and Particle Removal

**DOI:** 10.3390/membranes12030255

**Published:** 2022-02-23

**Authors:** Ali Sayegh, Simon Merkert, Joscha Zimmermann, Harald Horn, Florencia Saravia

**Affiliations:** 1 Engler-Bunte-Institut, Water Chemistry and Water Technology, Karlsruhe Institute of Technology, Engler-Bunte-Ring 9, 76131 Karlsruhe, Germany; ali.sayegh@kit.edu (A.S.); simon.merkert@web.de (S.M.); harald.horn@kit.edu (H.H.); 2 Institute of Catalysis Research and Technology, Karlsruhe Institute of Technology, Hermann-Von-Helmholtz-Platz 1, 76344 Eggenstein-Leopoldshafen, Germany; joscha.zimmermann@kit.edu; 3 DVGW-Research Center at the Engler-Bunte-Institut, Water Chemistry and Water Technology, Karlsruhe Institute of Technology, Engler-Bunte-Ring 9, 76131 Karlsruhe, Germany

**Keywords:** hydrothermal-liquefaction wastewater, crossflow ultrafiltration, ceramic membranes, oil and particle removal

## Abstract

This study aims to evaluate the application of ceramic ultrafiltration membranes in the crossflow mode for the separation of particles and oil in water emulsions (free oil droplets and micelles) from hydrothermal-liquefaction wastewater (HTL-WW) from the hydrothermal liquefaction of municipal sewage sludge. The experiments were carried out using one-channel TiO_2_ membranes with pore sizes of 30, 10 and 5 nm. The results showed that the highest stable permeability could be achieved with a membrane-pore size of 10 nm, which experienced less fouling, especially through pore blockage, in comparison to the two other pore sizes. Instead of observing an increase in the permeability, the application of a higher feed temperature as well as backwash cycles led to a clear increase in irreversible fouling due to the presence of surfactants in the HTL-WW. Among several physical and chemical cleaning methods, alkaline cleaning at pH 12 proved to be the most efficient in removing fouling and maintaining stable performance on a long-term basis. Ceramic-membrane ultrafiltration can be considered as an adequate first-stage treatment of real HTL wastewater.

## 1. Introduction

Fossil resources are the traditional sources for the production of fuels, but their availability is limited. To find an alternative for fossil fuels, interest in biofuel production is rising. Hydrothermal liquefaction (HTL) of biomass is one of the emerging technologies which valorizes different wet biological feedstocks for the production of biocrude as a blended stock of liquid transportation fuel [1]. Typical HTL process parameters range between temperatures of 250–450 °C and pressures of 100–300 bar. Water remains either in its liquid state or a relatively dense supercritical state under these pressure and temperature conditions. The advantage of HTL over other liquefaction processes is that the energy-intensive drying step is excluded [2].

In addition to the desired biocrude, an aqueous-phase byproduct is produced, which is the so-called HTL wastewater (HTL-WW). HTL-WW is enriched with organic substances and cannot be discharged into the environment without being treated [3]. This issue can be considered a bottleneck for the wide application of HTL processes in the future. Several technologies have been investigated for the valorization of HTL-WW, such as anaerobic digestion, adsorption (of phenolics and nitrogenous compounds) on activated carbon and hydrothermal gasification (HTG) and catalytic hydrothermal gasification (CHG) [4,5,6]. However, these techniques face limitations, such as the toxicity of HTL-WW to anaerobic compounds such as phenols, which originates from highly concentrated inhibitory compounds, the blockage of adsorbent media in activated carbon by particulates, as well as the high temperature, pressure and cost of the catalysts used in CHG [7,8,9]. HTL-WW could be treated via the membrane-filtration process, which is becoming an attractive solution due to its low energy consumption and higher filtration flux [3].

The driving force in most membrane processes is the pressure difference across the membrane [10]. The pressure-driven membrane processes are microfiltration (MF), ultrafiltration (UF), nanofiltration (NF) and reverse osmosis (RO), which differ in the separation properties of the membranes [11]. Lyu et al. was successful in treating a model solution of HTL-WW with NF and RO combined [12]. However, this would not be applicable for real HTL-WW without a pretreatment step, since it would lead to colloidal organic fouling followed by a rapid decrease in the permeate flux and membrane selectivity [13]. As an example, the pretreatment of wastewater resulting from olive-oil production via MF showed an efficient retention of suspended materials and production of a clarified permeate that was further treated via RO in order to separate the dissolved substances from water [14]. Among the above mentioned pressure-driven membrane processes, UF is one of the most effective pretreatment methods for oily wastewater in comparison with the traditional separation methods (mechanical separation, filtration, and chemical de-emulsification) [15].

UF can operate in static or dynamic modes. In static (dead-end) mode, the liquid part of the feed penetrates the membrane up to its complete volume, leaving behind the components that are larger than the membrane pores. The flow is perpendicular to the membrane surface, which leads to the formation of a cake layer from the retained particles on this surface. This cake layer creates a resistance to the flow, hence reducing the permeate flux over time. Consequently, frequent backwashing and chemical cleaning are indispensable in the removal of fouling and the restoration of the system flux and/or pressure to its original value [16,17]. In the case of the dynamic (crossflow) mode, one part of the feed solution passes through the membrane surface (permeate), while the other part flows tangentially along the membrane surface (concentrate). Unlike the static operation, the cake layer formed as a result of this operation does not increase at a steady rate. This is because the shear forces created as a part of the parallel feed flow prevents a steady cake-layer buildup by detaching the particles deposited on the membrane surface [18].

UF membranes can be categorized by their material into organic and inorganic membranes. Common commercial UF membranes are made of organic polymer materials such as polyethersulfone (PES), polyvinylidene fluoride (PVDF), polyacrylonitrile (PAN), poly-sulfone (PS) and polyvinyl chloride (PVC) [19]. In order to be applied to oil–water separation, additional hydrophilic coatings (e.g., catechol/chitosan) are being tested on polymeric membranes (e.g., PVDF) [20]. On the other hand, ceramic membranes are made out of inorganic materials such as alumina (Al_2_O_3_), zirconia (ZrO_2_), titania (TiO_2_), silicon carbide (SiC) and glass (SiO_2_) [21]. Ceramic membranes are adequate for the separation of oil emulsions in wastewaters because of the hydrophilic properties of oxide membranes [22]. In addition, they are tolerant against extremely low and high pH values, are thermally stable and can resist temperatures of up to several hundred degrees [21], which make them attractive to the treatment of HTL-WW. In brief, ceramic membranes present some advantages in comparison to polymeric membranes depending on the application [21,23]. However, ceramic membranes have a significantly higher production cost than polymeric membranes, and hence are used in harsh environments where polymeric membranes are inadequate, such as corrosive and high-temperature environments.

The two major challenges in water treatment via membrane processes are the permeate-product quality, based on the rejection of targeted solutes, and the membrane-fouling impact on the reduction in the filtration-process efficiency [24]. Fouling results in a permeate flux decline over time when the process is operated under constant-transmembrane-pressure (TMP) conditions, or in a TMP increase under constant-flux conditions [25,26]. Fouling can be categorized as reversible or irreversible. Irreversible fouling is the most problematic because it produces a long-term flux decline, which cannot be fully recovered by hydraulically cleaning the membrane [27]. The main mechanisms of fouling are (1) the adsorption of partially rejected matter within the membrane pores leading to their narrowing, (2) the blocking of pores by particles of a size range similar to that of the pores, and (3) the cake formation via the accumulation of completely rejected particulate matter on top of the membrane surface [28]. In addition, fouling can be grouped into three types: (1) biofouling, which is enhanced in the presence of transparent exopolymer particles (TEPs), (2) inorganic fouling (scaling) and (3) organic fouling [29,30]. Since bacterial growth is limited by the high ammonia concentration and the presence of recalcitrant organic compounds (e.g., phenols), biofouling is not expected to play a major role. According to the composition of the HTL-WW, organic fouling is the main contributor to fouling formation, thereby reducing membrane performance.

Sayegh et al. dealt with the pretreatment of real HTL-WW via submerged-membrane filtration [31]. To our knowledge, no studies have been published so far regarding the pretreatment of real HTL-WW with crossflow filtration. The aim of this study is to investigate the performance of ceramic ultrafiltration membranes, in crossflow mode, for the pretreatment of real HTL-WW. In addition to the oil and particle retention, the target is to determine the right conditions for UF to reduce the effect of fouling and maintain high and stable permeability. The parameters tested were the membrane-pore size, feed temperature and application of backwash intervals during filtration, in addition to physical and chemical cleaning.

## 2. Materials and Methods

### 2.1. Feed Solution

**Figure 1:**
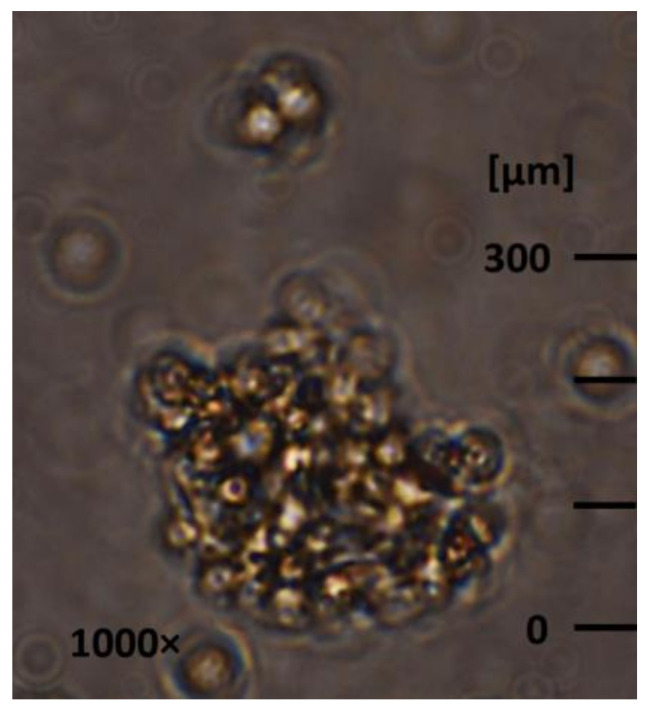
Free oil droplets and oil agglomerates in HTL-WW.

The feed solution used in this study was the wastewater of a hydrothermal-liquification process of sewage sludge, also called HTL-WW. This HTL-WW had a pH value of 9, a total-suspended-solids (TSS) concentration of 0.8 g/L and total-organic-carbon (TOC) concentration of 35 g/L (Table 1). The oil-in-water emulsion represented a significant part of the suspended agglomerates. As seen in Figure 1, free oil droplets (up to 50 µm diameter) could be visualized in HTL-WW, which can gather at the static state and form agglomerates ≥300 µm. On the other hand, the emulsified-oil size could even be as small as 10 nm if trapped inside micelles, which were stabilized with anionic, cationic and non-ionic surfactants found in the liquid (Table 1) [32]. The formation of micelles depends on the critical-micelle concentration of surfactants, which is defined as the minimum needed concentration of surfactant to form micelles. More information regarding the production of HTL-WW and its characteristics can be found elsewhere [31].

### 2.2. Membranes

In this work, ceramic membranes from the company Inopor (Germany) were used for particle retention and oil recovery. All experiments were carried out in crossflow operation according to the in-out principle. Three ultrafiltration membranes were used, which had active layers of TiO_2_ with pore sizes of 30 nm, 10 nm and 5 nm. All membranes had a supportive layer of α-Al_2_O_3_ with a pore size of 3 µm. Each membrane consisted of a single channel with an inner diameter of 7 mm and an outer diameter of 10 mm. The unified membrane length was 250 mm, of which 224 mm was active.

### 2.3. Filtration Setup

**Figure 2:**
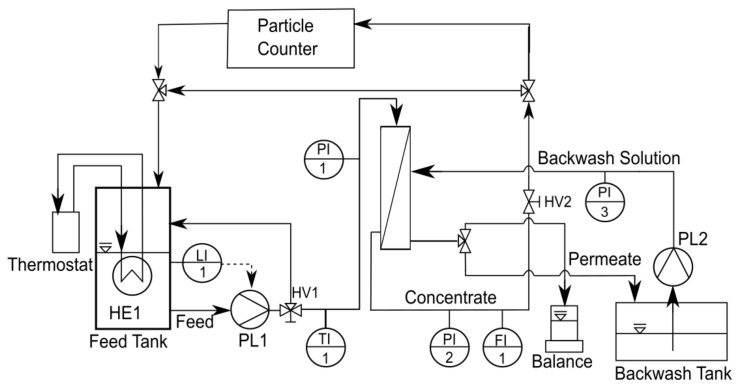
Filtration setup.

Figure 2 shows the experimental setup of crossflow ultrafiltration. The feed tank was filled with HTL-WW up to 10 L. Temperature was controlled using a thermostat with the aid of the heat exchanger (HE1) submerged in the feed solution. The level indicator (LI1) was needed to protect the pump. HTL-WW was pumped via a rotary lube pump (PL1) (Xylem, Norderstedt, Germany). The hand valve HV1 was used to regulate the ratio between the return flow to the feed tank and the input flow into the membrane vessel. Feed pressure and temperature were measured upstream of the membrane vessel by the pressure indicator PI1 and the temperature indicator TI1, respectively. The membrane vessel had two outputs: the concentrate and the permeate.

The concentrate pressure and flow rate were measured via the pressure indicator PI2 and the flow indicator FI1, respectively. The hand valve HV2, downstream of the membrane vessel, was used to control both pressure and flow rate. The particle counter (HACH, Düsseldorf, Germany) was used to measure, online, the particle-size distribution of the HTL-WW (concentrate) stream based on the number of particles per mL of liquid at discrete sizes of 2, 3, 5, 10, 15, 25, 50 and 100 µm. These measurements could help to understand the effect of filtration conditions (such as filtration time, temperature and flow rate) on the characteristics of feed HTL-WW during ultrafiltration.

The collection of the permeate depends on the filtration mode. If backwash was excluded, the permeate was continuously collected on a mass balance in order to measure the filtration flux. If backwash was included, the permeate was split evenly between the mass balance and the backwash tank. A filtration cycle lasted for 30 min, of which the last 30 s was a backwash mode. During the backwash mode, the permeate collected in the backwash tank was pumped via pump PL2 (Seko, Wiesbaden, Germany) into the permeate side of the membrane. The backwash pressure could be observed using the pressure indicator PI3.

The data acquisition of pressure, temperature, flow rate and permeate flux as well as the control of the analog valves was performed using the LabVIEW software (National Instruments, Austin, TX, USA).

Six experiments of 1-week periods in addition to critical-flux measurements were executed under a fixed-flow velocity of 0.5 m/s. The flow velocity was chosen based on 2 boundary conditions. Turbulent conditions in the membrane channels must be ensured in order to allow deformation, sliding and detachment of the oil droplets adhering to the membrane surface [33]. On the other hand, high flow velocities can lead to the elongation of a single circular oil droplet emulsified in the solution into elliptical shape, followed by deformation into a dumbbell-shaped particle before breaking [34]. The breaking of big oil emulsions/particles into smaller ones due to the crossflow velocity was observed in this work using the online particle counter, which could show decreases in numbers of particles with diameters of 10, 15 and 25 µm and increases in the particles with diameters of 2 and 3 µm over time (Supplementary Figure S1). To minimize the effect of deformation on membrane-pore blockage, higher flow velocities were not used.

**Figure 3:**
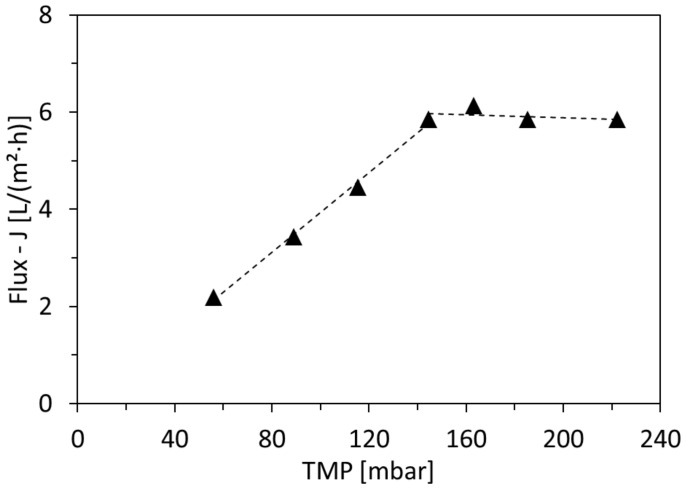
Critical-flux and critical-TMP determination for 5 nm pore size membrane.

The aim of the critical-flux measurements was to determine the critical TMPs and, subsequently, the feed pressures applied to the long-term experiments. Le Clech et al,. introduced 7 methods for this measurement [35]. According to the aim of this study, the method applied is based on how the flux changes upon the stepwise increase in TMP. The pressure was raised every 30 min to permit flux stabilization, until the flux became pressure independent. From each measuring interval, the average flux was plotted as a function of the average TMP, as seen later in Figure 3. The critical pressure is defined as the intercept of the plateau with the linear flux variation [36].

The long-term experiments were operated under variable conditions of membrane-pore size and feed temperature with or without backwash cycles as shown in Table 2.

### 2.4. Cleaning Methods

Membrane cleaning aims to restore the permeability, which degrades as a result of fouling. Membrane cleaning can be categorized as physical, chemical, biological/biochemical or physico-chemical [37]. In this work, all membranes were cleaned after each long-term experiment or critical-flux measurement. After experiments 1, 2 and 3, the membranes underwent cleaning procedures of two types. First, physical cleaning was performed with demineralized water in three steps (up to one hour each): (1) high crossflow velocity (1.5 m/s), (2) high temperature (50 °C) and (3) backwash cycles. This was followed by chemical cleaning, which consisted of two steps: (1) alkaline cleaning (pH 12) followed by (2) acid cleaning (pH 2). The cleaning detergents used were Atec_2610 (Atec Neu-Ulm, Neu-Ulm, Germany), which is an alkaline membrane cleaner mainly consisting of sodium hydroxide and tetrasodium ethylenediamine tetra-acetate, and Atec_AC_3027 (Atec Neu-Ulm, Neu-Ulm, Germany), which is an acid membrane cleaner mainly consisting of nitric acid and phosphoric acid. After experiments 4, 5 and 6, only chemical cleaning was executed. Physical cleaning after experiments 1, 2 and 3 was performed in the filtration system (Figure 2), while chemical cleaning was applied in a separate system to prevent corrosive effects of the cleaning detergents on the metal parts of the filtration system. For chemical cleaning, the crossflow velocity was 0.16 m/s and the duration of cleaning was several hours.

### 2.5. Analytical Methods

Cationic, anionic, and non-ionic surfactants were measured using test kits LCK 331, LCK 332 and LCK 333, respectively, from Hach Lange, Germany. The assessment of total-organic-carbon (TOC) concentrations was performed using a TOC Analyzer (Shimadzu TOC-V CPN) (Shimadzu, Kyoto, Japan). Microscopic imaging was performed using a Leica DMR microscope (Leica Microsystems, Mannheim, Germany). A Zeta Seizer Nano ZS (Malvern Panalytical, Malvern, UK) was used to measure, offline, the particle-size distribution of the permeate samples (with a measuring range of 0.6 nm to 6000 nm), in order to determine the particle size of the largest volume fraction (explained later in the results section).

**Figure 4:**
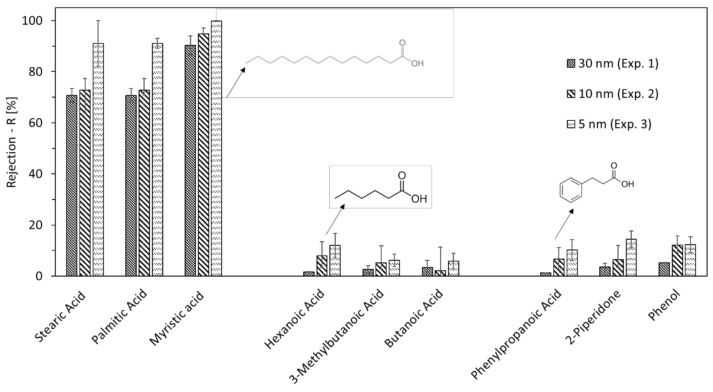
Rejection of long-chain fatty acids (stearic acid, palmitic acid and myristic acid), short-chain fatty acids (hexanoic acid, 3-methylbutanoic acid and butanoic acid) and cyclic compounds (phenylpropanoic acid, 2-piperidone and phenol) for UF membranes-pore sizes of 30, 10 and 5 nm in experiments 1, 2 and 3, respectively.

The chemical composition of the HTL-WW was determined by gas chromatography (GC) (Agilent 6890N) (Agilent, Santa Clara, CA, USA) coupled with mass spectroscopy (MS) (Agilent 5973) (Agilent, Santa Clara, CA, USA). Due to the relatively high pH value and the occurrence of emulsions, a specific sample preparation was needed. In the first step, the samples were acidified with 0.5 wt.% sulfuric acid to a pH between 3–4 and extracted with chloroform (ratio 2:1). Subsequently, 200 µL of the extract was mixed with 50 µL of a chloroform solution including 1000 ppm of pyridine. To derivatize the acidic components, 50 µL *N*,*O*-Bis(trimethylsilyl)trifluoroacetamide (BSTFA) + 1% Trimethylchlorosilane (TCMS) was added and heated to 70 °C for 1 h. Selected-ion-monitoring (SIM) was applied to the detected components (Figure 4) in the chloroform extract. The compounds were externally calibrated, and distribution coefficients (K_D_) were determined based on a model solution. The quantifier and qualifier ions and coefficients are listed in the Supplementary Table S1.

### 2.6. Data Interpretation

The instantaneous permeation flux J (L/(m^2^·h)) was determined as follows:

(1) $$J=\frac{\mathrm{dV}}{A\cdot \mathrm{dt}}$$

where dV, A, and dt represent the (differential) total volume (L) of permeate collected over time period (dt), the effective permeation area (m^2^) and the operating time (h), respectively.

The membrane permeability P (L/m^2^·h·bar) is defined as follows:

(2) $$P=\frac{J}{\mathrm{TMP}}$$

where J is the instantaneous permeation flux (L/(m^2^·h)) and TMP is the transmembrane pressure (bar).

The apparent rejection R (%) for a given component x by the membrane is calculated as follows:

(3) $$R_{X}=\frac{C_{f}-C_{p}}{C_{f}}⬝100\%$$

where C_f_ (g/L) is the feed concentration and C_p_ (g/L) is the permeate concentration

## 3. Results and Discussion

### 3.1. Critical-Flux Measurements

Critical-flux measurements were initially applied in order to define the filtration conditions that prevent rapid fouling. The measurements were applied with a gradual increase in pressure every 30 min. For all tested membranes, the flux (Equation (1)) increased linearly as a function of the pressure until the critical flux was reached, after which it stabilized. An example of the critical-flux measurement is shown in Figure 3 and the results are summarized in Table 3. The results show that at 25 °C, the three membranes of pore sizes 30, 10 and 5 nm had critical-flux values of 8.3, 6.6 and 6.1 L/m^2^·h, respectively. This was expected as the increase in pore size allows for higher flow rates through the pores. The critical flux of the 10 nm pore size membrane was also checked at 40 °C, and it was remarkable that it measured 5.2 L/m^2^·h, which is lower than that at 25 °C. This shows that higher feed temperature can promote fouling, which will be further discussed in the following sections.

Based on these results, it was decided to operate the long-term experiments under the low feed pressure of 70 mbar for membranes-pore sizes of 30 and 10 nm and 100 mbar for the membrane-pore size of 5 nm in order to prevent rapid fouling.

### 3.2. Permeate Quality

The TOC rejection (Equation (3)) of the membrane-pore sizes of 30 nm, 10 nm and 5 nm was 3%, 6% and 15%, respectively. This low rejection is directly related to the organic constituents of HTL-WW. For example, a comparison was made between some detected organic compounds (listed in Table 1) by distributing them into three groups: long-chain fatty acids, short-chain fatty acids, and cyclic compounds. As shown in Figure 4, all three long-chain fatty acids (stearic acid, palmitic acid and myristic acid) had rejections higher than 70% (90% in the case of the 5 nm pore size). On the other hand, the rejection of short-chain fatty acids and cyclic compounds did not exceed 15%.

**Figure 5:**
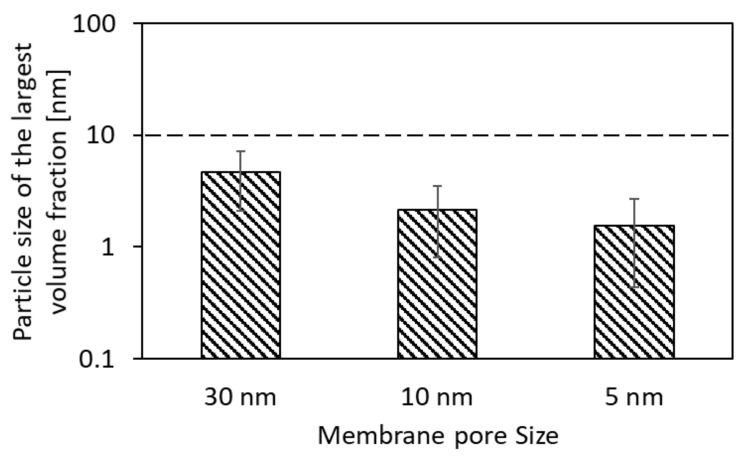
Permeate quality based on the particle size of the largest volume fraction of produced permeates for UF membrane-pore sizes of 30, 10 and 5 nm from experiments 1, 2 and 3, respectively.

Although the long-chain fatty acids have the highest molecular weights among the three groups, their rejection cannot be based on their size since the membrane pores are much larger and cannot retain them as free molecules. Nevertheless, the solubilities of long-chain fatty acids in water are much lower compared to the other two groups, meaning they will mainly be present as part of the emulsified oil in HTL-WW. Emulsified oil in water can be present in many sizes, but the smallest form is 10 nm and occurs when it is trapped inside micelles. As shown in Figure 5, the particle sizes of the largest volume fraction of the collected permeates from ultrafiltration with membrane-pore sizes of 30 nm, 10 nm and 5 nm were 4.6 nm, 2.1 nm and 1.6 nm, respectively. This shows a significant rejection of micellar oil emulsions, since the presence of particles greater than 10 nm in the permeate is not significant.

The aim of applying particle-size-distribution measurements was to determine the largest particles in the permeate and to check if it exceeded the limit of 10 nm. Since the particle-size distribution is measured based on the dynamic-light-scattering method (used in Zeta Seizer Nano ZS), large particles might interfere with the measurement of smaller ones. However, the goal of the measurement was solely to characterize the largest particles passing through the membrane. This can be represented by “the particle size of the largest volume fraction”, which is not affected by the presence of small particles. The particle size of largest volume fraction was determined from several particle-size-distribution measurements that were applied offline to permeate samples on a daily basis (an example is shown in the Supplementary Figure S2). It represents the dominating particle size in the permeate with the highest volume ratio among all the present particle sizes, which makes it the most relevant for analyzing the permeate quality. 

In addition to the size of a micelle, its charge plays a significant role in its rejection. This is shown in Table 4, as more than 85% of the anionic surfactants were retained, since the active membrane surface holds a negative charge at the pH value of 9. Only up to 31% of the cationic surfactants were retained since the electrostatic interaction with the membrane surface leads to the adsorption of the positively charged surfactants on the membrane surface followed by their penetration into the permeate by the applied pressure. Neutral surfactants were barely retained, except when the 5 nm membrane was used (R = 18%). In this case, these surfactants can adsorb on the inner part of the membrane-pore surface through hydrophilic and electrostatic interactions [38], thus leading to narrowing of the filtration channels and faster degradation of the filtration flux.

### 3.3. Optimal Membrane-Pore Size

The decrease in permeability is an indication of membrane fouling. This decline takes place due to the accumulation of foulants on the membrane surface, inside the membrane pores, or both [39]. The degree of fouling depends on the operating parameters, feed stream and membrane characteristics [40]. The membrane-pore size plays a significant role in minimizing or maximizing each fouling mechanism, especially for feed solutions containing oil emulsions. To investigate this issue, membranes with different pore sizes were used.

**Figure 6:**
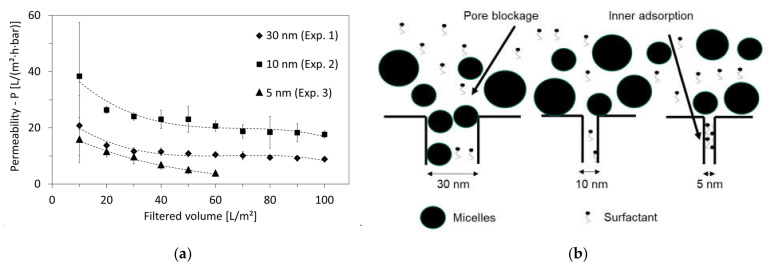
(**a**) Comparison of permeability of experiments performed using the membrane-pore sizes of 30 nm (experiment 1), 10 nm (experiment 2) and 5 nm (experiment 3) (applied TMP is 70 mbar for membrane-pore sizes of 30 and 10 nm and 100 mbar for the membrane-pore size of 5 nm) and (**b**) assumed pore blockage by micelle and (mainly cationic and non-ionic) surfactant adsorption on the inner walls of membrane pores.

The first comparison was made between the pore sizes of 30 nm and 10 nm from experiments 1 and 2, respectively. Although the membrane with the 30 nm pore size achieved a higher critical flux as shown in Table 3 (8.3 L/(h·m^2^) compared to 6.6 L/(h·m^2^) for the 10 nm pore size), it experienced a lower permeability (Equation (2)) after a filtered volume of 10 L/m^2^, as shown in Figure 6a. In both experiments 1 and 2, there existed an initial decrease in permeability until it stabilized, after a filtered volume of 100 L/m^2^, at approximately 9 L/m^2^·h·bar and 18 L/m^2^·h·bar for the 30 nm and 10 nm pore sizes, respectively.

The typical initial fouling is standard pore blockage. A possible reason for the sharper drop in permeability for the 30 nm pore size in comparison to that of the 10 nm is the presence of micelles with diameters smaller than 30 nm, which can close the pore entrance by standard blockage, as shown in Figure 6b. This is not the case for the 10 nm pore size, since the micelles have, in general, diameters ≥10 nm [32].

As a result, the performance of the 10 nm membrane was better, and was then compared with the smaller pore size of 5 nm from experiment 3. The latter showed the lowest permeability values from the beginning of the filtration, as well as a steady (linear) decrease until a filtered volume of 60 L/m^2^, after which the membrane was completely blocked. The continuous performance degradation of the 5 nm membrane-pore size could be due to the high adsorption of large molecules such as the non-ionic surfactants on the inner walls of the membrane pores, as shown in Figure 6b. Adsorption occurred for all membranes and narrowed their pores, but its effect on fouling was apparently the highest for the smallest pore size of 5 nm. This can be supported by the relatively higher rejection of non-ionic surfactants by the 5 nm pore size, in comparison with the 30 nm and 10 nm pore sizes, as shown earlier in Table 4.

As a result of these findings, the membrane with a pore size of 10 nm was selected for further experiments.

### 3.4. Optimal Operation Conditions

Permeability restoration after membrane fouling is indispensable to the efficient application of the membrane on a long-term basis. One option for the recovery of permeability is the application of counterflow (backwash). Backwash is applied for the removal of reversible fouling, which mainly consists of non-adherent deposited species on the membrane surface. However, it is not efficient against fouling matter that is adsorbed on the inner walls of the membrane pores, which is therefore considered irreversible fouling [41].

**Figure 7:**
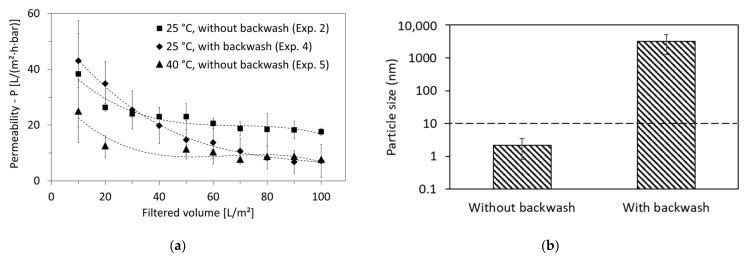
(**a**) Comparison of permeability of experiments performed using the membrane-pore size of 10 nm at different operating conditions of feed temperature and backwash cycles: 25 °C without backwash (experiment 2), 25 °C with backwash (experiment 4) and 40 °C without backwash (experiment 5) and (**b**) permeate quality based on the particle size of largest volume fraction of produced permeates for UF membrane of pore size 10 nm without and with backwashing from experiments 2 and 4, respectively.

Backwash intervals were therefore introduced in experiment 4, aiming to improve the permeability of the 10 nm-pore-size membranes. As shown in Figure 7a, backwash improved the permeability until a filter volume of 30 L/m^2^. After that, the permeability decline was faster than without backwash and was down to 7 L/m^2^·h·bar at a filtered volume of 100 L/m^2^ (in comparison with 18 L/m^2^ from experiment 2 performed without backwash).

Earlier studies found that during backwash, small-molecular-weight foulants present in the permeate are capable of infiltrating the membrane pores and leading to their blockage, especially if excessive backwash is used [42]. As shown in Figure 7b, the permeate of the 10 nm membrane-pore size had a particle size of the largest volume fraction of 2.1 ± 1.3 nm if no backwash was applied. Additionally, the filtration showed poor rejection of cationic (31%) and non-ionic (5%) surfactants (Table 4). Thus, it can be confirmed that small-molecular-weight foulants were present in the permeate and might have had a critical effect on membrane fouling if backwash had been applied, in addition to blocking the membrane from the permeate side.

In the case of backwash, Figure 7b shows the particle size of the largest volume fraction in the produced permeate in the range of 3.2 ± 2.0 µm. The formation of these particles in the permeate during backwash can be triggered by the concentration polarization of surfactants on the permeate side of the membrane. Since backwash is applied in dead-end mode and at a relatively high pressure (up to 2 bar), surfactant concentrations on the membrane surface on the permeate side can exceed the CMC, leading to the formation of micelles. These micelles could be formed inside the membrane pores of the supportive layer (which had 3 µm pore size) and on its surface. This was confirmed by the visual observation of a fouling layer on the external walls of the membranes, as shown in Supplementary Figure S3.

As a result, filtration without backwash cycles was decided to be the better option.

To increase the HTL-WW permeability of the membrane, experiments were performed by lowering the density at higher temperatures. Thus, experiment 5 was performed with identical conditions to experiment 2 except that the feed temperature was elevated from 25 °C to 40 °C. However, the results presented in Figure 7a show a faster degradation of permeability at higher temperatures, which reached 7 L/m^2^·h·bar at a filtered volume of 100 L/m^2^ (in comparison with 18 L/m^2^ for 25 °C).

This can be explained by the initial fouling formation, which might have happened at the beginning (before 10 L/m^2^ volume was filtered), since the decrease in density could trigger high initial fluxes. In addition, the critical-flux measurements (Table 3) show that irreversible fouling at 40 °C starts at a lower flux (5.2 L/m^2^·h) than at 25 °C (6.6 L/m^2^·h). This indicates that the fouling can increase due to this rise in temperature. Mohajeri et al. [43] investigated the effect of temperature on the CMC of surfactants. It was shown, among the three non-ionic surfactants investigated in the study (Polysorbate-20, Polysorbate-40 and Polysorbate-80), that CMC drops along with the increase in temperature from 25 °C to 40 °C [43]. This can also be the case for surfactants present in HTL-WW. More micelles form after the temperature elevation by 15 °C, which can eventually enhance initial fouling. This is supported by particle-size-distribution measurements that were performed online via the particle counter at the beginning and end of experiments 4 and 5. The Supplementary Figure S1 shows that for all the measured sizes, the number of particles in the HTL-WW feed were higher at 40 °C (experiment 5) in comparison to 25 °C (experiment 4).

In addition to organic fouling, the scaling of CaCO_3_ and struvite (MgNH_4_PO_4_·6H2O) at elevated temperatures may also lead to lower permeability. Moreover, Schork et al., showed that the presence of calcium ions upon the filtration of a sodium alginate solution with ceramic membranes enhanced the formation of dense and compact fouling layers, which could only partially detach after backwash [44]. However, these phenomena were not thoroughly investigated because the concentrations of calcium and magnesium in HTL-WW were very low.

### 3.5. Optimal Cleaning Method

Due to fouling in experiment 2, the pure-water permeability (PWP) of the 10 nm-pore-size membrane decreased by 57% (from 211 L/m^2^·h·bar to 90 L/m^2^·h·bar). To compensate for this decrease and reduce fouling, several physical and chemical cleaning methods were tested using demineralized water and cleaning agents, respectively, and summarized in Table 5.

As a first cleaning step, the increase in the crossflow velocity (CFV) by three times from 0.5 m/s to 1.5 m/s showed no improvement of the PWP. Raising the feed temperature by two times from 25 °C to 50 °C led to an improvement of 5% in the second step of treatment; however, it decreased by 3% upon applying backwash in the third treatment step. Hence it could be understood that physical cleaning leads only to a minor improvement of PWP.

On the other hand, chemical cleaning with an alkaline cleaner achieved the greatest recovery, since the PWP increased by 26% between the third and fourth cleaning steps. Increasing the pH value to 12 helped to increase the repulsive forces between the negatively charged membrane surface and the fouled organic compounds. This pH increase supports the hydrolysis and ionization of the carboxyl groups and hydroxyl groups, eventually leading to the detachment of the fouling layer [45]. The last cleaning step was chemical cleaning with an acid solution at pH 2. This step improved the PWP only by 5%. This means that inorganic fouling (scaling) played only a minor role.

Similar trends of PWP improvement were observed among cleaning the fouled membrane-pore sizes of 30 nm and 5 nm after experiments 1 and 3, respectively. Among all the cleaning steps, only the alkaline cleaning showed a significate improvement of PWP, which increased by 22% and 64% for the membrane-pore sizes of 30 nm and 5 nm, respectively. As a result, alkaline cleaning is recommended in order to maintain adequate performance of crossflow UF of HTL-WW using ceramic membranes. In addition, since the efficient cleaning method was chemical but not physical, it could be deduced that irreversible fouling plays a major role in HTL-WW permeability reduction through UF membranes. This is not the case for the treatment of other wastewaters, such as swimming-pool water, where reversible fouling is significant [46].

**Figure 8:**
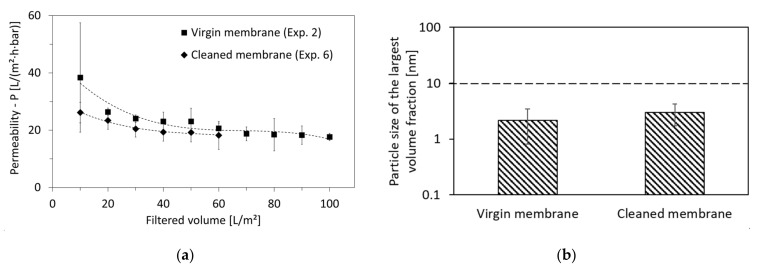
(**a**) Comparison of permeability of HTL-WW and (**b**) permeate quality based on the particle size of largest volume fraction of produced permeates, both in experiments performed using the membrane-pore size of 10 nm on a virgin membrane (experiment 2) and on a cleaned membrane after being used for several weeks (experiment 6).

For the cleaning of the 10 nm membrane in experiments 4, 5 and 6, the cleaning was only carried out chemically using the alkaline/acidic sequence. The efficiency of this cleaning could be confirmed when comparing the permeability of experiments 2 and 6. Both experiments had identical conditions, except that the membrane was fresh when used for experiment 2, while it had been used for several weeks before experiment 6 took place. As shown in Figure 8a, the difference in permeability between both experiments is in the acceptable range of 10–15%. Even the quality of the permeate remained consistent between both experiments, which can be seen through the similar particle size of largest volume fraction in Figure 8b. This shows the robustness of the membrane material against the HTL-WW constituents, the cleaning agents, and the change in temperature.

## 4. Conclusions

This study showed that ceramic-membrane ultrafiltration employed under crossflow operation was efficient in retaining particulate matter and oil emulsions in HTL-WW. The membrane with a pore size of 10 nm was effective in maintaining a stable filtration with a permeability of 18 L/m^2^·h·bar, to be operated at room temperature, without backwash cycles and with a feed pressure of 70 mbar. Several physical and chemical cleaning methods were investigated and showed no notable augmentation of physical cleaning in recovering the PWP. However, a significant improvement of PWP after alkaline chemical cleaning was achieved, which increased by 26 % for the membrane with the 10 nm pore size. In conclusion, crossflow UF can be adapted as a first-stage filtration prior to further treatment of real HTL-WW (e.g., reverse osmosis or membrane distillation).

## Figures and Tables

**Table 1:** Composition of HTL-WW.

Parameter	Value (g/L)	Parameter	Value (g/L)
TSS	0.8	Stearic Acid	0.3
TOC	35	Benzenpropanoic Acid	1.0
Non-ionic surfactants	1.3	2-Piperidone	4.1
Cationic surfactants	0.4	Butanoic Acid	0.7
Anionic surfactants	0.3	3-Methylbutanoic Acid	1.3
Mystiric Acid	0.1	Hexanoic Acid	0.4
Palmitic Acid	0.5	Phenol	0.3

**Table 2:** Overview of conditions of the experiments used for this study.

Experiment No.	Time of Operation [d]	Membrane-Pore Size [nm]	Temperature [°C]	Backwash Cycles [yes/no]
1	7	30	25	no
2	7	10	25	no
3	7	5	25	no
4	8	10	25	yes
5	8	10	40	no
6 *	3	10	25	no

* Experiment 6 is exclusively to check the cleaning procedure efficiency after optimization of all parameters.

**Table 3:** Critical flux and TMP for different membrane-pore sizes and temperatures.

Pore Size [nm]	Temperature [°C]	Critical TMP [mbar]	Critical Flux [L/(m^2^·h)]
30	25	90	8.3
10	25	75	6.6
5	25	150	6.1
10	40	60	5.2

**Table 4:** Rejection (R) of surfactants in experiments 1, 2 and 3 for membrane-pore sizes of 30, 10 and 5 nm, respectively (5% standard deviation).

Surfactants’ Rejection—R [%]			
Membrane-Pore Size	Anionic	Cationic	Non-Ionic
30 nm	85	20	3
10 nm	>90	31	5
5 nm	87	30	18

**Table 5:** Recovery of pure-water permeability (PWP) after experiment 2 by the aid of several physical and chemical cleaning methods (PWP measured 211 L/m^2^·h·bar and 90 L/m^2^·h·bar before and after experiment 2, respectively; recovery before cleaning was 43%; cleaning steps 1 to 5 were performed sequentially).

Cleaning Step	Time of Operation (h)	Cleaning Method	PWP (L/m^2^·h·Bar)	Recovery (%)
1	1	Rising CFV (1.5 m/s)	89	42
2	1	Rising temperature (50 °C)	100	47
3	1	Applying backwash	93	44
4	24	Alkaline cleaning (pH 12)	149	70
5	24	Acid cleaning (pH 2)	157	75

## Data Availability

The data that support the findings of this study are available on request from the corresponding author.

## Supplementary Materials

The following supporting information can be downloaded at: https://www.mdpi.com/article/10.3390/membranes12030255/s1, Figure S1: Online particle-size distribution of HTL-WW for both feed temperatures of 25 °C and 40 °C at the beginning and end of the experiments 4 and 5, respectively; Figure S2: Example on determination of particle size of largest volume fraction (here: 3.7 nm) from the particle-size distribution of a permeate sample measured offline; Figure S3: Fouling on the permeate side of the membrane after backwash cycles (experiment 4); Table S1: Quantifier and qualifier ions and coefficients.
