# Supplementary material for: Treatment of Hydrothermal-Liquefaction Wastewater with Crossflow UF for Oil and Particle Removal
Source: Membranes (Basel). 2022 Feb 23;12(3):255. doi: 10.3390/membranes12030255 (PMC8951593; doi:10.3390/membranes12030255)
Supplement: Supplementary file 1 [file membranes-12-00255-s001.zip › membranes-1601275-supplementary.pdf]

membranes

MDPI

Supplementary Materials

Treatment of Hydrothermal-Liquefaction Wastewater with Crossflow UF for Oil and Particle Removal

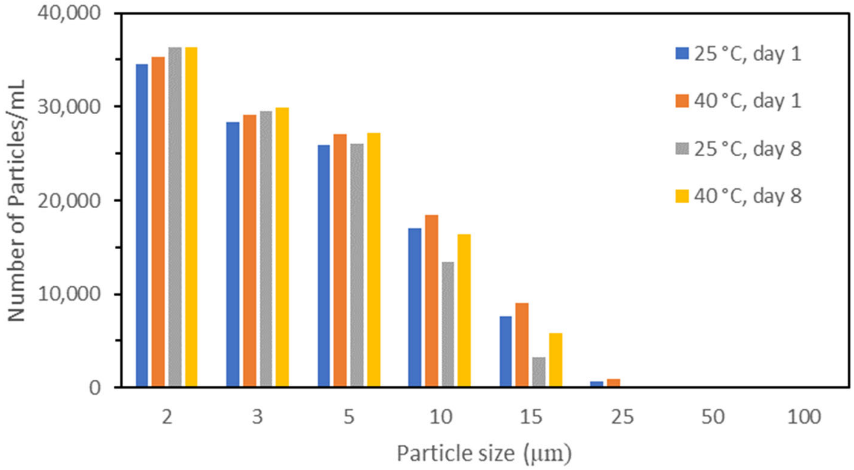

Figure S1. Online particle size distribution of HTL-WW for both feed temperatures of 25 °C and 40 °C at the beginning and end of the experiments 4 and 5, respectively.

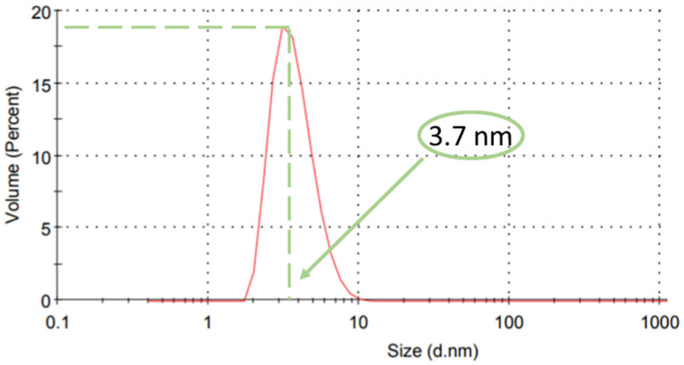

Figure S2. Example on determination of particle size of largest volume fraction (here: 3.7 nm) from the particle size distribution of a permeate sample measured offline.

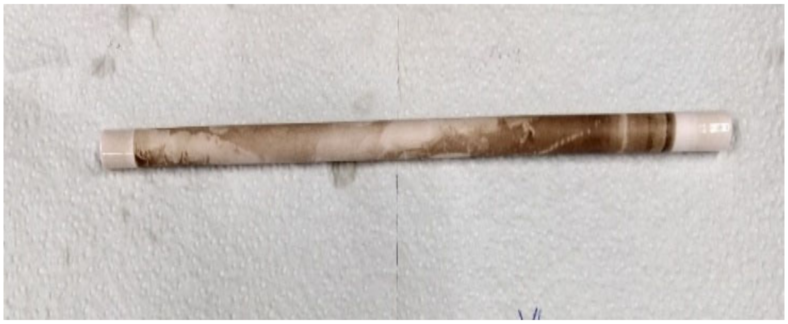

Figure S3. Fouling on the permeate side of the membrane after backwash cycles (experiment 4).

Table S1. Quantifier (Quant) and qualifier (Qual) ions and coefficients.

Compound (TMS derivative)	Quant - Ion	Qual - Ion 1	Qual - Ion 2	Calibration curve	R^2	KD coefficient
Butyric Acid	145.0	117.0	75.0	y = 5573.678605x	0.9846	0.2245
3-Methylbutanoic acid	159.0	117.0	75.0	y = 5477.467552x	0.9843	0.3905
Hexanoic acid	173.0	117.0	75.0	y = 4861.313633x	0.9845	0.5267
2-Piperidinone	156.0	171.0	170.0	y = 5437.271723x	0.9870	0.1939
Phenol	166.0	151.0	-	y = 4875.999941x	0.9861	0.4007
Benzenepropanoic acid	207.0	222.0	104.0	y = 2960.176371x	0.9874	0.5519
Myristic acid	285.0	129.0	117.0	y = 2781.936124x	0.9718	0.8552
Palmitic Acid	313.0	129.0	117.0	y = 2351.042887x	0.9688	0.8598
Stearic Acid	341.0	129.0	117.0	y = 2306.811049x	0.9780	0.8608
